# Supplementary material for: Identification of photoperiod-induced specific miRNAs in the adrenal glands of Sunite sheep (Ovis aries)
Source: Front Vet Sci. 2022 Jul 22;9:888207. doi: 10.3389/fvets.2022.888207 (PMC9354845; doi:10.3389/fvets.2022.888207)
Supplement: Supplementary file 1 [file Data_Sheet_1.ZIP › Supplementary file/miRDeep2 file.zip!/miRDeep2 file/Know_Structure/LP42A1/oar-mir-30a.pdf]

miRBase precursor : oar-mir-30a
Total read count : 126119
oar-miR-30a-5p read count : 118035
oar-miR-30a-3p read count : 8084
remaining reads : 0

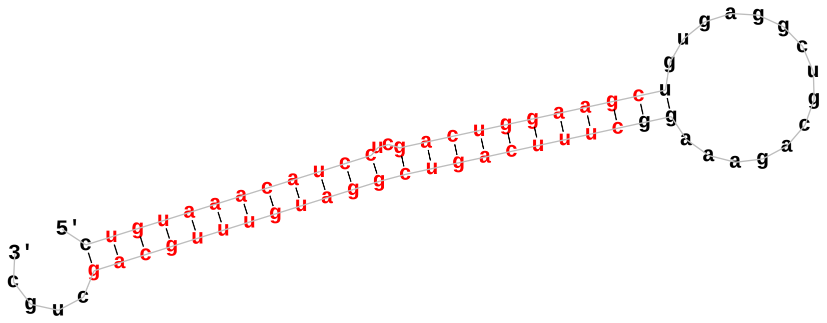

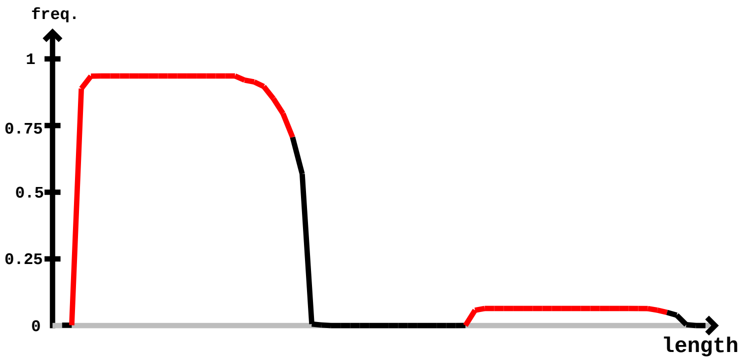

oar-miR-30a-3p

oar-miR-30a-5p

5'- cuguaaacauccucgacuggaagcugugaggcugcagaaaggcuuucagucggauguuugcagcugc -3' exp

((((((((((((..((((((((((((...............)))))))))))))))))))))))....	reads	mm	sample
cuguaaacauccuUgacu...	3	1	seq
cuguaaacauccuUgacug..	1	1	seq
cuguaaacauccucgacugg...	5	0	seq
cuguaaacauccuUgacugg...	3	1	seq
cuguaaacauccucgacugga..	26	0	seq
cuguaaacauccucgacuggaa...	20	0	seq
cuguaaacauccucgacuggaag..	14	0	seq
cuguaaacauccuUgacuggaag..	1	1	seq
cuguaaacauccucgacuggaagc...	23	0	seq
cuguaaacauccucgacuggaagcu..	39	0	seq
cuguaaacauccuUgacuggaagcu..	31	1	seq
.uguaaacauccuUgacu...	1719	1	seq
.uguaaacauccucgacu...	230	0	seq
.uguaaacauccucgacug..	213	0	seq
.uguaaacauccuUgacug..	641	1	seq
.uguaaacauccuUgacugg...	1150	1	seq
.uguaaacauccucgacugg...	1015	0	seq
.uguaaacauccucgacugga..	4622	0	seq
.uguaaacauccuUgacugga..	1046	1	seq
.uguaaacauccuUgacuggaa...	1189	1	seq
.uguaaacauccucgacuggaa...	5828	0	seq
.uguaaacauccuUgacuggaag..	3725	1	seq
.uguaaacauccucgacuggaag..	7446	0	seq
.uguaaacauccuUgacuggaagc...	2874	1	seq
.uguaaacauccucgacuggaagc...	14357	0	seq
.uguaaacauccuUgacuggaagcu..	39581	1	seq
.uguaaacauccucgacuggaagcu..	26097	0	seq
.uguaaacauccucgacuggaagcug...	37	0	seq
.uguaaacauccuUgacuggaagcug...	93	1	seq
.uguaaacauccucgacuggaagcugu..	16	0	seq
.uguaaacauccuUgacuggaagcugu..	38	1	seq
..guaaacauccuUgacug..	1	1	seq
..guaaacauccuUgacugg...	4	1	seq
..guaaacauccuUgacugga..	14	1	seq

oar-miR-30a-3p

oar-miR-30a-5p

cuguaaacauccucgacuggaagcugugaggcugcagaaaggcuuucagucggauguuugcagcugc

..guaaacauccucgacugga..	3	0	seq
..guaaacauccucgacuggaa...	4	0	seq
..guaaacauccuUgacuggaa...	6	1	seq
..guaaacauccucgacuggaag..	12	0	seq
..guaaacauccuUgacuggaag..	41	1	seq
..guaaacauccucgacuggaagc...	29	0	seq
..guaaacauccuUgacuggaagc...	60	1	seq
..guaaacauccucgacuggaagcu..	227	0	seq
..guaaacauccuUgacuggaagcu..	4947	1	seq
..guaaacauccucgacuggaagcug...	3	0	seq
..guaaacauccuUgacuggaagcug...	315	1	seq
..guaaacauccuUgacuggaagcugu..	199	1	seq
...uaaacauccuUgacugga..	1	1	seq
...uaaacauccuUgacuggaagc...	1	1	seq
...uaaacauccucgacuggaagcu..	18	0	seq
...uaaacauccuUgacuggaagcu..	42	1	seq
...uaaacauccuUgacuggaagcug...	12	1	seq
...uaaacauccucgacuggaagcug...	1	0	seq
...uaaacauccuUgacuggaagcugu..	11	1	seq
....aaacauccucgacuggaagcu..	1	0	seq
....gcuuucagucggauguu.........	1	0	seq
....gcuuucagucggauguuuAc......	4	1	seq
....gcuuucagucggauguuuAca.....	14	1	seq
....gcuuucagucggauguuuAcag....	9	1	seq
....gcuuucagucggauguuuAcagc...	5	1	seq
....cuuucagucggauguuu........	19	0	seq
....cuuucagucggauguuug.......	12	0	seq
....cuuucagucggauguuuA.......	17	1	seq
....cuuucagucggauguuugc......	124	0	seq
....cuuucagucggauguuuAc......	588	1	seq
....cuuucagucggauguuuAca.....	920	1	seq
....cuuucagucggauguuugca.....	99	0	seq
....cuuucagucggauguuugcag....	308	0	seq
....cuuucagucggauguuuAcag....	924	1	seq
....cuuucagucggauguuugcagc...	1239	0	seq
....cuuucagucggauguuuAcagc...	2858	1	seq
....cuuucagucggauguuugcagcu..	102	0	seq
....cuuucagucggauguuugcagcug.	1	0	seq
....uuucagucggauguuuA.......	1	1	seq
....uuucagucggauguuuAc......	32	1	seq
....uuucagucggauguuugc......	14	0	seq
....uuucagucggauguuuAca.....	14	1	seq
....uuucagucggauguuugca.....	2	0	seq
....uuucagucggauguuugcag....	25	0	seq
....uuucagucggauguuuAcag....	33	1	seq
....uuucagucggauguuugcagc...	176	0	seq
....uuucagucggauguuuAcagc...	209	1	seq
....uuucagucggauguuugcagcu..	319	0	seq
....uuucagucggauguuugcagcug.	9	0	seq
....uucagucggauguuuAcagc...	1	1	seq
....uucagucggauguuugcagc...	5	0	seq
